# Disease-Specific Contribution of Pulvinar Dysfunction to Impaired Emotion Recognition in Schizophrenia

**DOI:** 10.3389/fnbeh.2021.787383

**Published:** 2022-02-14

**Authors:** Antígona Martínez, Russell H. Tobe, Pablo A. Gaspar, Daniel Malinsky, Elisa C. Dias, Pejman Sehatpour, Peter Lakatos, Gaurav H. Patel, Dalton H. Bermudez, Gail Silipo, Daniel C. Javitt

**Affiliations:** ^1^Nathan Kline Institute for Psychiatric Research, Orangeburg, NY, United States; ^2^College of Physicians and Surgeons, Columbia University, New York, NY, United States; ^3^Department of Psychiatry, Biomedical Neurosciences Institute, IMHAY, University of Chile, Santiago, Chile; ^4^Mailman School of Public Health, Columbia University, New York, NY, United States; ^5^New York State Psychiatric Institute, New York, NY, United States

**Keywords:** fMRI, emotion, schizophrenia, autism, visual, subcortical, pulvinar, faces

## Abstract

One important aspect for managing social interactions is the ability to perceive and respond to facial expressions rapidly and accurately. This ability is highly dependent upon intact processing within both cortical and subcortical components of the early visual pathways. Social cognitive deficits, including face emotion recognition (FER) deficits, are characteristic of several neuropsychiatric disorders including schizophrenia (Sz) and autism spectrum disorders (ASD). Here, we investigated potential visual sensory contributions to FER deficits in Sz (*n* = 28, 8/20 female/male; age 21–54 years) and adult ASD (*n* = 20, 4/16 female/male; age 19–43 years) participants compared to neurotypical (*n* = 30, 8/22 female/male; age 19–54 years) controls using task-based fMRI during an implicit static/dynamic FER task. Compared to neurotypical controls, both Sz (*d* = 1.97) and ASD (*d* = 1.13) participants had significantly lower FER scores which interrelated with diminished activation of the superior temporal sulcus (STS). In Sz, STS deficits were predicted by reduced activation of early visual regions (*d* = 0.85, *p* = 0.002) and of the pulvinar nucleus of the thalamus (*d* = 0.44, *p* = 0.042), along with impaired cortico-pulvinar interaction. By contrast, ASD participants showed patterns of increased early visual cortical (*d* = 1.03, *p* = 0.001) and pulvinar (*d* = 0.71, *p* = 0.015) activation. Large effect-size structural and histological abnormalities of pulvinar have previously been documented in Sz. Moreover, we have recently demonstrated impaired pulvinar activation to simple visual stimuli in Sz. Here, we provide the first demonstration of a disease-specific contribution of impaired pulvinar activation to social cognitive impairment in Sz.

## Introduction

Social cognitive deficits are a core feature of schizophrenia (Sz) (Fernandes et al., 2018) and autism spectrum disorders (ASD) and contribute to impaired functional outcome (Mancuso et al., 2011; Bishop-Fitzpatrick et al., 2017). One important aspect of social functioning is the ability to rapidly and accurately perceive facial expressions. Impaired face-emotion recognition (FER) has been extensively reported in Sz (Edwards et al., 2002; Kohler et al., 2010) and ASD (Harms et al., 2010; Uljarevic and Hamilton, 2013; Tobe et al., 2016) however the underlying neuronal substrates of these deficits are not fully understood and, indeed, may arise from differential underlying neural pathologies (Foss-Feig et al., 2017). Over recent years, the contribution of sensory-processing deficits to cognitive impairments has been increasingly appreciated (Javitt and Freedman, 2015; Koshiyama et al., 2021), including the potential role of dysfunction within subcortical components of the afferent visual streams (Koshiyama et al., 2018; Martinez et al., 2019). Here, we utilize functional magnetic resonance imaging (fMRI) to evaluate the contributions of impaired early sensory processing to FER impairments in Sz. Data were collected as well from both neurotypical and ASD comparison groups to assess the specificity and magnitude of observed activation deficits in Sz.

During normative brain function, FER is supported by activation of specific components of the “social brain”(Adolphs, 2009), which includes structures along both the dorsal and ventral visual-cortical pathways (Allison et al., 2000; Haxby et al., 2000; Pitcher et al., 2011). These pathways receive retinal information from the lateral geniculate nucleus (LGN), which projects to primary visual cortex (V1). The dorsal pathway receives its primary input from the magnocellular geniculostriate pathway and is specialized for rapid detection of low spatial-frequency and motion information. Key dorsal structures include motion-sensitive mid-temporal regions (MT, MST). In Sz, differential deficits in magnocellular processing have been reported and related to potentially impaired patterns of sensory gain and functions of the N-methyl-D-aspartate-type glutamate receptors (NMDAR) (reviewed in Javitt and Freedman, 2015). Moreover, impairments in magnocellular function correlate with behavioral measures of impaired FER in Sz, supporting the involvement of this pathway in social cognition (Martinez et al., 2018, 2019; Marosi et al., 2019). By contrast, the ventral visual stream receives predominant input from the subcortical parvocellular system and is specialized for slower but higher-resolution processing of stimulus details. Key targets of the ventral stream include visual area V4 and the fusiform face complex (FFC).

Along with the ventral and dorsal pathways, the presence of an anatomically and functionally distinct third pathway specialized for social perception and comprising the superior temporal sulcus (STS) region, has recently been proposed (Pitcher and Ungerleider, 2021). The STS region has been reliably associated with processing biological motion signals (Kilts et al., 2003; Sato et al., 2004; Deen et al., 2015) including dynamic social cues such as the changeable aspects of facial features (eyes, lips). Impaired STS activation has been documented in Sz but the basis for the deficit remains unknown (Kim et al., 2011; Mier et al., 2014, 2017; Kronbichler et al., 2017; Matsumoto et al., 2018).

In addition to the cortical system, humans retain an evolutionarily old retinotectal system that mediates non-conscious affective processing *via* amygdala, superior colliculus and the pulvinar nucleus of the thalamus (PulN) (reviewed in Tamietto and Morrone, 2016). In addition to mediating retinogeniculate input into visual cortex, PulN also mediates cortico-cortical interactions between successive brain regions within the dorsal and ventral stream pathways (e.g., Bridge et al., 2016), and is the site of greatest NMDAR density within primate thalamus (Ibrahim et al., 2000).

PulN is anatomically divided into discrete, functionally differentiated subdivisions (e.g., Bourgeois et al., 2020; Guedj and Vuilleumier, 2020). For example, the “visual pulvinar,” consisting of its inferior (PI) and lateral (PL) subdivisions, has dense connections with early visual sensory regions (Guedj and Vuilleumier, 2020) and likely plays a modulatory role in visual information processing (de Souza et al., 2020). In addition, projections from PI specifically innervate motion sensitive regions surrounding area MT, especially MST (Kaas and Baldwin, 2019), and also serve as drivers to secondary areas of visual cortex (e.g., V2) and as modulators to V1 (de Souza et al., 2020). On the other hand, medial pulvinar (PM) is considered multimodal and is primarily coupled with prefrontal and temporal regions including STS (Homman-Ludiye and Bourne, 2019) and may play a unique role in processing emotional information (reviewed in Arend et al., 2015).

Here, we evaluate whole-brain fMRI activation patterns during FER in Sz, relative to both neurotypical individuals and ASD. Inclusion of ASD participants is based on a previous study involving simple visual stimuli in which we observed a divergent pattern of disturbance within early visual areas and PulN relative to Sz patients, despite similar magnitude of FER impairment (Martinez et al., 2019). We hypothesized that, in Sz, deficits within FER-related higher tier visual regions (e.g., STS) would be driven significantly by impaired activation of both early visual regions (e.g., V1, MST) and PulN as well as by impaired cortico-pulvinar interactions. Moreover, we hypothesized that deficit patterns would be differential across Sz and ASD participants despite similar levels of behavioral impairment, suggesting disorder-specific pathophysiological mechanisms underlying social cognitive impairments in neuropsychiatric populations.

## Materials and Methods

### Participants

Seventy-eight participants took part, including 28 participants (age range 21–54 years) diagnosed with schizophrenia (Sz) using the Structured Clinical Interview for DSM-IV (First et al., 1994), 20 adults with autism spectrum disorder (ASD) (age range 19–43 years), confirmed by the Autism Diagnostic Observation Schedule, Second Edition, and 30 neurotypical controls (age range 19–54 years) (Table 1). All Sz participants were on a stable dose of antipsychotic medication. All participants had at least 20/22 corrected visual acuity on a Logarithmic Visual Acuity Chart. On average, Sz participants were older [*F*(1, 56) = 7.24, *p* = 0.009] and had lower IQ scores [*F*(1, 56) = 6.54, *p* = 0.013] than controls. All ASD participants and a subset of 19 Sz and 17 controls participated in our previous EEG/fMRI study of visual sensory dysfunction as reported in Martinez et al. (2019), which did not include data from the present paradigm. Participants were recruited from the central research database and volunteer recruitment pool at the Nathan Kline Institute for Psychiatric Research (NKI). The investigation was approved by the Nathan Kline Institute (NKI) institutional review board. Informed consent was obtained from all participants.

**TABLE 1:** Participant characteristics.

	CTL	SZ	ASD
Age	31.4 (9.8)	38.8 (10.1)**	28.9 (7.7)
Gender (F/M)	8/22	8/20	4/16
Years of education	14.6 (2.0)	11.8 (2.0)***	13.7 (2.5)
Participant SES	39.6 (11.5)	24.4 (6.3)***	33.7 (11.8)
Parental SES	46.1 (14.8)	38.3 (13.1)	49.2 (11.5)
IQ	103.1 (8.9)	96.7 (9.9)*	101.2 (9.0)
PSI	101.5 (12.3)	81.8 (12.7)***	–
POI	108.9 (16.1)	88.7 (15.7)***	–
Illness duration (years)	–	14.2 (9.1)	–
CPZ equiv. SZ	–	834.8 (629.1)	–
Anti-psychotic medication type		Typical (6)	
		Atypical (15)	
		Combination (7)	
ER-40	35.2 (1.9)	28.7 (5.5)***	30.4 (4.7)+++
PANSS (positive)	–	11.1 (4.1)	–
PANSS (negative)	–	19.3 (5.6)	–
ADOS-2 (Comm.)	–	–	4.7 (1.5)
ADOS-2 (Social Inter.)	–	–	8.3 (2.5)

CTL, Control; SZ, schizophrenia; ASD, autism spectrum disorder; CPZ, Chlorpromazine equivalents; PSI, Processing Speed Index; POI, Perceptual Organization Index; ER-40, Penn Emotion Recognition Task; PANSS, Positive and Negative Syndrome Scale; ADOS-2, Autism Diagnostic Observation Schedule, Second Edition, Communication (Comm.) and Social Interaction (Social Inter.) scores. Asterisks denote significant differences between CTL and SZ participants; plus signs denote differences between CTL and ASD participants (**/++p < 0.01; ***/+++p < 0.001). Standard deviations in parentheses. *p < 0.05.

### Paradigm

**FIGURE 1:**
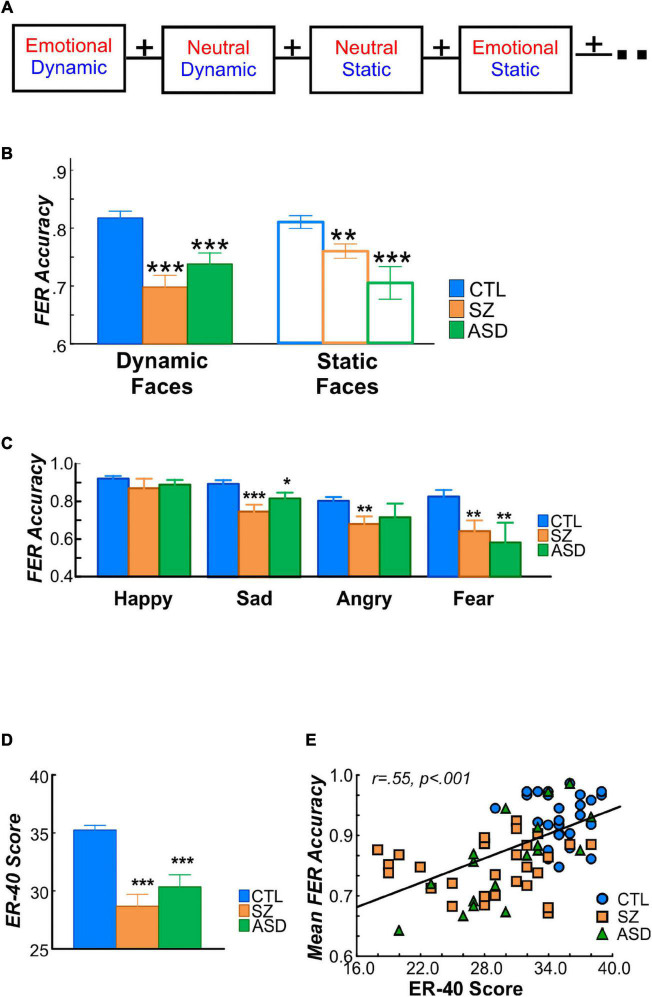
**(A)** Schematic of fMRI paradigm. A total of 20 blocks lasting 12 s each were delivered in random order in each of two fMRI scans. Each block consisted of faces expressing a single emotion (happy, sad, angry, fear) or neutral expression either dynamically or statically. **(B)** FER accuracy determined in the behavioral paradigm administered outside the scanner, for dynamic (filled bars) and static (open) faces in control (CTL; blue), schizophrenia (SZ; orange), and autism (ASD; green). Relative to the CTL group, FER accuracy was significantly lower in the SZ and ASD groups for both dynamic (left bars) and static (right) faces. In SZ patients, FER accuracy for dynamic faces was especially reduced, relative to control participants. **(C)** Mean FER accuracy as a function of face-emotion and (below) sample stimuli used for happy, sad, angry and fear emotions. FER accuracy did not differ overall as a function of face-emotion and group membership. **(D)** Groupwise scores on the Penn Emotion Recognition (ER-40) test. As expected, both SZ and ASD participants had significantly lower ER-40 scores compared to the CTL group, which, **(E)** were correlated with accuracy on the FER task. In all cases, significance of group differences is denoted by asterisks, relative to CTL (*p < 0.05; **p < 0.01;***p < 0.005).

Unique video clips of five actors (three male) dynamically expressing each of four emotions (happy, sad, angry, fearful) were selected from the University of Cambridge Mind Reading Emotions Library (adult level 6) (Golan et al., 2006). Five additional actors (two male) from the NKI community acted a neutral expression consisting of non-emotionally salient head/eye movements (left/right, up/down). Neutral videos were matched in size, resolution and luminance to emotion videos. For each video, representative single frames were extracted and used as corresponding static stimuli. Both dynamic and static stimuli were presented for 2 s each followed by a 400 ms interstimulus interval (ISI). In each of two ∼7.5-min fMRI scans, dynamic and static stimuli of a single emotion or neutral were delivered in 12-s blocks (5 stimuli per block), interleaved with 10-s of fixation-only. A total of ten blocks of static and 10 blocks of dynamic faces were presented in random order per scan (Figure 1A). Across both scans a total of 200 stimuli were delivered, 20 of each of four emotions plus neutral, either dynamic or static. To ascertain that participants were attending the stimuli, participants responded by button press to a single predesignated actor chosen randomly for each participant, irrespective of emotion or motion. The target actor appeared in ∼10% of all stimuli.

### Behavior Measures

A forced-choice behavioral task was administered following the fMRI scan using the same static and dynamic emotional face stimuli (80 stimuli total, 20 of each type; neutral faces were not included). As in the fMRI scan, each static/dynamic stimulus was presented for 2 s. After each presentation, subjects were prompted to press one of five buttons to indicate if the actor’s expression was (1) happy, (2) sad, (3) angry, (4) fearful, or (5) none of the above. Accuracy, as opposed to response time was emphasized. The trial ended when subjects responded. To compare findings from our FER paradigm with those from a validated and reliable measure of FER, the Penn Emotion Recognition test (ER-40) (Taylor and MacDonald, 2012) was also administered to all participants and its results compared to those of the present FER paradigm. The ER-40 uses forty color photographs of faces expressing four basic emotions—happiness, sadness, anger, or fear—plus neutral—with eight photographs for each category, presented in random order. Participants were instructed to choose the correct emotion from among the five listed choices (forced choice) by clicking a computer mouse as quickly as possible without sacrificing accuracy. Each image was displayed until a choice was made.

### Functional Imaging

Imaging took place on a Siemens 3T TiM Trio scanner. Two-hundred-twenty T2*-weighted echo-planar images (EPIs) (*TR* = 2,000 ms; *TE* = 30 ms; *FA* = 90°; FOV = 240 mm; slice thickness = 2.8 mm) were acquired on each of 36 contiguous slices in the axial plane. At least one high-resolution structural image of the entire brain was acquired from each participant using an MPRAGE sequence (*TR* = 2500 ms; *TE* = 3.5 ms; FOV = 256 mm, slice thickness = 1.0 mm, 192 slices).

Individual cortical surfaces were rendered from the high-resolution anatomical images using Freesurfer and registered to the std 0.141 fsaverage mesh (Fischl et al., 1999) with SUMA.^1^ The pulvinar and amygdala were derived individually using a Bayesian atlas-based automated segmentation methods (Saygin et al., 2017; Iglesias et al., 2018; Bocchetta et al., 2020) incorporated in Freesurfer. Functional data were preprocessed and analyzed using the Analysis of Functional NeuroImages (AFNI) software (Cox, 1996; Saad and Reynolds, 2012). Preprocessing consisted of concatenating data from two runs, removal of signal deviation >2.5 SDs from the mean (AFNI’s 3dDespike), temporal alignment, identification of motion outliers per run and scaling of blood-oxygen-level-dependent (BOLD) values to mean percent signal change (Taylor et al., 2018). For surface-based analyses, the data was spatially smoothed with a 6 mm full width at half maximum Gaussian kernel. Single-participant statistical analyses were conducted within the framework of the general linear model (GLM). The GLM model included regressors for each stimulus type (emotional dynamic, emotional static, neutral dynamic, neutral static) as well as regressors for the six motion parameters (three rotations, three translations) and their first derivatives, per run. Time points with large head motion between successive time points were censored. Surface-based analyses were carried out on the gray-matter ordinates of each individual cortical surface aligned to the Freesurfer 141-standard mesh. Cortical data was sampled to the Human Connectome Project multimodal cortical parcellation (HCP-MMP1.0) (Glasser et al., 2016), resampled to fsaverage, which delineates 180 brain parcels per hemisphere based on functional and structural properties. To assess activation of pulvinar and amygdala, analyses were conducted in the individual native-space volumes. Primary analyses involved the entire pulvinar. In secondary analyses, beta parameters were extracted from pulvinar subdivisions and tested separately.

**FIGURE 2:**
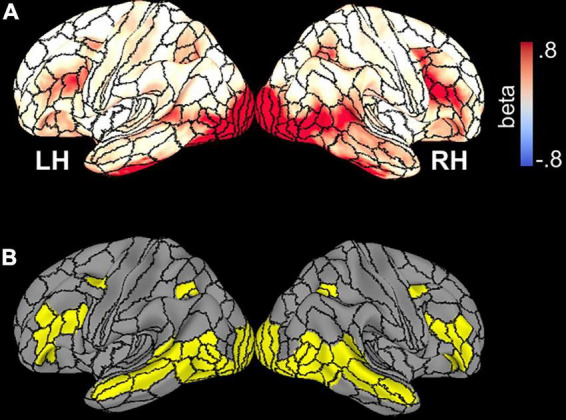
**(A)** Whole-brain beta parameter maps of activation elicited by all stimuli and across all participants, superimposed on the template brain with borders of HCP parcels demarcated. **(B)** Thirty-five parcels with significant activation collapsed across subjects and face stimuli.

To avoid issues related to circularity in data analysis (Kriegeskorte et al., 2009), activated parcels were first identified by an unpaired *t*-test of mean activation (vs. 0), collapsed over all stimuli, across all seventy-eight participants thresholded at an (uncorrected) *p*-value of 0.001 (Figure 2A). This analysis defined a mask consisting of 35 bilateral parcels (70 parcels total) (Figure 2B) in the HCP MMP1.0 parcellation atlas which was used in subsequent analyses of functional data. Table 2 lists each parcel.

**TABLE 2:** Mean beta parameter values in each of the 35 HCP-MMP1.0 (Glasser et al., 2016) parcels shown in Figure 2B for the control (CTL), schizophrenia (SZ), and autism (ASD) groups.

	HC	SZ	ASD	HC vs. SZ		HC vs. ASD		ASD vs. SZ	
				*F*(1, 56)	*p*	*F*(1, 48)	*p*	*F*(1, 46)	*p*

V1	0.56 (0.32)	0.30 (0.31)	0.37 (0.45)	10.41	0.002*	3.16	0.082	0.45	0.506
V2	0.18 (0.52)	0.28 (0.77)	0.70 (0.48)	0.33	0.569	12.52	0.001*	4.58	0.038*
V3	0.36 (0.27)	0.31 (0.30)	0.42 (0.34)	0.50	0.484	0.38	0.541	1.30	0.260
V4	1.47 (0.59)	1.07 (0.40)	1.45 (0.81)	2.78	0.101	1.83	0.183	0.00	0.917
V8	0.63 (0.40)	0.66 (0.40)	0.83 (0.64)	0.07	0.788	1.93	0.171	1.38	0.247
FFC	1.32 (0.57)	1.00 (0.53)	0.89 (0.53)	4.98	0.030*	6.99	0.011*	0.42	0.522
PIT	1.35 (0.51)	1.31 (0.63)	1.34 (0.85)	0.06	0.811	0.00	0.977	0.02	0.886
VVC	0.40 (0.29)	0.51 (0.32)	0.44 (0.46)	1.62	0.208	0.10	0.758	0.39	0.535
MST	0.50 (0.18)	0.33 (0.21)	0.44 (0.23)	4.10	0.022*	0.09	0.915	4.54	0.016*
LO2	1.03 (0.51)	1.11 (0.44)	1.03 (0.68)	0.47	0.494	0.00	0.976	0.26	0.614
MT	0.37 (0.22)	0.46 (0.37)	0.51 (0.28)	1.50	0.222	3.80	0.058	0.17	0.679
PH	0.40 (0.29)	0.49 (0.38)	0.40 (0.34)	0.96	0.332	0.00	0.998	0.66	0.422
V4t	0.75 (0.41)	0.94 (0.44)	0.89 (0.55)	3.07	0.085	1.12	0.295	0.12	0.727
FST	0.00 (0.15)	−0.03(0.18)	0.00 (0.21)	0.44	0.508	0.00	0.983	0.29	0.593
FEF	0.24 (0.17)	0.29 (0.24)	0.12 (0.21)	0.77	0.383	4.24	0.044*	6.27	0.016*
STSda	0.10 (0.15)	0.12 (0.20)	0.06 (0.19)	0.17	0.685	0.91	0.344	1.25	0.270
STSdp	0.31 (0.20)	0.17 (0.21)	0.13 (0.20)	7.39	0.009*	7.25	0.010*	0.24	0.728
STSvp	0.04 (0.15)	−0.04(0.31)	−0.01(0.19)	1.55	0.219	1.08	0.304	0.13	0.724
STSva	0.09 (0.17)	0.15 (0.34)	−0.01(0.16)	0.88	0.352	3.30	0.075	4.14	0.048*
FOP5	0.10 (0.14)	0.09 (0.22)	0.19 (0.20)	0.02	0.901	3.45	0.069	2.37	0.131
TE2p	0.25 (0.31)	0.31 (0.43)	0.22 (0.42)	0.39	0.535	0.08	0.774	0.52	0.473
PHT	0.15 (0.22)	0.17 (0.29)	0.09 (0.23)	0.07	0.786	0.91	0.346	1.06	0.308
STV	0.27 (0.16)	0.17 (0.21)	0.22 (0.27)	4.56	0.037*	0.85	0.360	0.48	0.494
TPOJ1	0.39 (0.15)	0.23 (0.21)	0.24 (0.26)	12.56	0.001*	6.78	0.012*	0.06	0.820
TPOJ2	0.35 (0.25)	0.34 (0.28)	0.28 (0.27)	0.00	0.958	0.69	0.410	0.52	0.476
TPOJ3	0.15 (0.20)	0.21 (0.26)	0.18 (0.18)	0.85	0.360	0.19	0.664	0.22	0.638
LIPd	0.25 (0.21)	0.27 (0.24)	0.24 (0.29)	0.04	0.845	0.03	0.872	0.09	0.767
IP1	0.19 (0.24)	0.13 (0.24)	0.18 (0.26)	1.16	0.287	0.03	0.856	0.56	0.457
IP0	0.08 (0.14)	0.19 (0.28)	0.07 (0.29)	3.21	0.079	0.02	0.894	1.80	0.186
45	0.14 (0.14)	0.09 (0.19)	0.11 (0.27)	1.08	0.303	0.27	0.607	0.05	0.816
IFJa	0.42 (0.25)	0.43 (0.33)	0.39 (0.37)	0.01	0.918	0.12	0.736	0.14	0.713
IFJp	0.36 (0.21)	0.40 (0.33)	0.35 (0.29)	0.24	0.626	0.05	0.830	0.30	0.585
IFSp	0.31 (0.19)	0.24 (0.24)	0.26 (0.29)	1.29	0.260	0.48	0.493	0.06	0.818
IFSa	0.17 (0.15)	0.14 (0.23)	0.13 (0.23)	0.24	0.630	0.56	0.458	0.05	0.819
p9-46v	0.17 (0.14)	0.15 (0.23)	0.13 (0.18)	0.10	0.755	0.84	0.363	0.19	0.662
Amyg.	0.20 (0.08)	0.39 (0.37)	0.25 (0.33)	7.58	0.008*	1.39	0.244	1.89	0.175
PulN	0.09 (0.08)	0.03 (0.17)	0.10 (0.14)	4.35	0.042*	0.26	0.614	2.91	0.095

Standard deviations in parentheses. F- and p-values for the main effect of group membership in the ANOVAs contrasting CTL vs. SZ, CTL vs. ASD, and ASD vs. SZ. Shaded parcels had a main effect of group membership. Values for subcortical regions, amygdala (Amyg.) and pulvinar (PulN) are given in last two rows. *p < 0.05.

### General Statistics

Mean beta parameter estimates were extracted from individual parcels and entered into an omnibus repeated measures analysis of variance (ANOVA) collapsing over all parcels identified as having significant across-group activation (*n* = 35 parcels per hemisphere, Table 2). Diagnostic group (control, Sz, ASD) was included as a between-subject factor. Face-motion type (dynamic, static) and face-emotion type (emotional, neutral) were included as within-subject factors. To minimize concerns regarding multiple comparisons, follow-up tests on individual parcels were conducted only if the initial group X parcel interaction was significant. Pre-planned subcortical regions (PulN, amygdala) were evaluated in separate ANOVAs with factors group, face-motion and face-emotion.

Effect sizes of between-group differences were calculated using Cohen’s *d* (mean/std dev).

The interrelationship between behavioral measures of FER and cortical/subcortical activation patterns was assessed by analysis of covariance (ANCOVA) with group membership as the categorical factor. Follow up ANCOVAs were conducted in a sequential fashion, for each significant covariate effect obtained in the prior analysis. In each case, only a single ANCOVA was performed for the region being tested as the dependent variable with all remaining covariates. The covariate x group interaction was used to evaluate group differences in the relationship between covariates.

All statistics were two-tailed with preset α level for significance of *p* < 0.05.

### Mediation Analyses

Based on our prior findings (Martinez et al., 2019), we used exploratory linear mediation analyses to explore the role of PulN subdivisions and cortical activation patterns in Sz patients. Analyses were conducted within SPSS26^2^ using the PROCESS macro (Hayes, 2013). A three-variable path model (model 4) was used to examine the predictor-outcome relationship between interrelated regions with impaired activation in Sz (relative to controls) and the potential mediating role of each PulN subdivision (PL, PI, PM). As per standard conventions, the link between the predictor and mediator variable is referred to as path *a*, and that between the mediator and the outcome (controlling for the predictor), is path *b*. The overall predictor-outcome relationship is effect *c*, and the direct effect, after controlling for the mediator is, *c′*. The indirect (mediation) effect is the product of a*b and tests the significance of *c – c′*. Statistical significance of indirect pathways, reflecting the impact of mediation, was evaluated using a non−parametric bootstrap approach with 10,000 replication samples to obtain a 95% confidence interval (CI) (Preacher and Hayes, 2008). The mediation effects were considered statistically significant if the bootstrapped 95% CI did not include zero.

## Results

### Behavior

Overall, FER accuracy was significantly lower in both Sz [*F*(1, 56) = 15.02, *p* < 0.001] and ASD participants [*F*(1, 48) = 7.67, *p* = 0.009] compared to controls but did not differ between Sz and ASD [*F*(1, 46) = 0.03, *p* = 0.863] participants (Figure 1B).

Across groups, FER accuracy was equivalent for dynamic and static faces [*F*(1, 75) = 0.314, *p* = 0.576], however, the group x face-motion interaction was significant [*F*(2, 75) = 8.26, *p* < 0.001], reflecting relatively greater deficits for processing dynamic faces in Sz patients compared to controls [*F*(1, 56) = 11.70, *p* = 0.001; *d* = 1.4 dynamic faces; *d* = 0.82 static faces]. Consequently, mean FER accuracy for dynamic vs. static faces was significantly lower in the Sz group [*t*(54) = −2.65, *p* = 0.010] but did not differ within either the ASD [*t*(38) = 1.01, *p* = 0.317] nor control [*t*(58) = 0.55, *p* = 0.584].

FER accuracy did not differ overall as a function of specific face-emotion type [*F*(6, 146) = 0.84, *p* = 0.541] (Figure 1C).

As expected, ER-40 scores were also lower in both Sz [*F*(1, 56) = 36.58, *p* < 0.001] and ASD [*F*(1, 48) = 24.57, *p* < 0.001] subjects compared to control participants (Figure 1D). Further, across subjects, ER-40 scores significantly predicted mean accuracy on the FER task [*F*(1, 74) = 8.22, *p* = 0.005; *R*^2^ = 0.407] (Figure 1E).

Lastly, behavioral performance on the identity recognition task during fMRI was marginally worse in Sz [*F*(1, 56) = 3.76, *p* = 0.058] and significantly worse in ASD [*F*(1, 48) = 4.52, *p* = 0.039] compared to controls.

### Between-Group Functional Magnetic Resonance Imaging Activation Differences

#### Cortical Surface

An initial omnibus analysis was carried out across all parcels in the mask (listed in Table 2) in order to test the null hypothesis that there were no significant activation differences across groups. The null hypothesis was falsified by the finding of a significant group x parcel interaction [*F*(68, 80) = 1.78, *p* = 0.007]. By contrast, the main effect of group membership was non-significant [*F*(2, 73) = 0.106, *p* = 0.899]. These findings were interpreted as indicating that activation of some, but not all, parcels differed significantly in activation across groups (Supplementary Table 1).

Follow-up analyses were therefore conducted to determine which parcels most contributed to the significant interaction effect observed in the omnibus test. The goal of these analyses was to identify regions that were most likely to contribute to between-group differences in behavioral task-performance. Therefore, these analyses were not considered to increase family-wise error rates.

**FIGURE 3:**
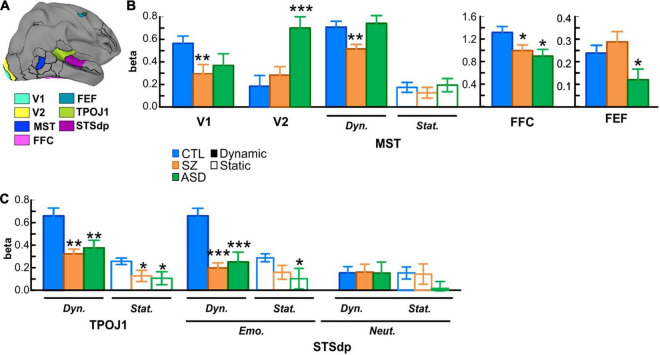
**(A)** Localization of key parcels with overall group differences: early visual areas V1 and V2; medial superior temporal cortex (MST); fusiform face complex (FFC); frontal eye fields (FEF); temporo-parietal junction (TPOJ1); and dorsal posterior superior temporal sulcus (STSdp). **(B)** Mean activation (beta parameter) extracted from each key parcel for the control (CTL; blue bars), schizophrenia (SZ; orange bars), and autism (ASD; green bars) groups. In parcels with a face-motion effect, activation to dynamic (solid bars) and static (open bars) faces are plotted separately. Asterisks indicate a significant difference in SZ or ASD groups relative to CTL. **(C)** Activation in the STS parcels (TPOJ1, STSdp) showing differential activation patterns for dynamic/static, emotional (Emo.)/neutral (Neut.) faces in STSdp.

In these protected follow-up analyses, significant main effects of group membership were obtained in seven parcels (V1,V2, MST, FFC, TPOJ1, STSdp, FEF) encompassing early visual areas [V1: *F*(2, 75) = 4.38, *p* = 0.016; V2: *F*(2, 75) = 4.49, *p* = 0.014]; motion-sensitive, medial superior temporal cortex [MST: *F*(2, 75) = 3.95, *p* = 0.023], fusiform face complex [FFC: *F*(2, 75) = 3.62, *p* = 0.032]; frontal eye fields [FEF: *F*(2, 75) = 3.63, *p* = 0.031] and the posterior superior-temporal cortex including the temporo-parietal junction [TPOJ1: *F*(2, 75) = 6.88, *p* = 0.002] and the dorsal posterior bank of the superior temporal sulcus [STSdp: *F*(2, 75) = 3.16, *p* = 0.048] (Table 2, highlighted parcels; Figure 3A).

When compared independently to the control group, patients with Sz had reduced mean activation in early visual [V1: *F*(1, 56) = 10.41, *p* = 0.002, *d* = 0.85] and motion-sensitive [MST: *F*(1, 56) = 4.10, *p* = 0.022, *d* = 0.76] parcels. ASD participants, in contrast, showed significantly higher activation of early visual regions [V2: *F*(1, 48) = 12.52, *p* = 0.001, *d* = 1.03] as well as reduced activity in prefrontal cortex [FEF: *F*(1, 48) = 4.24, *p* = 0.045, *d* = 0.59] relative to control subjects (Figure 3B).

In parallel with these divergent activation patterns, convergent deficits were observed in the pSTS parcels with reduced mean activation in both Sz [STSdp: *F*(1, 56) = 5.81, *p* = 0.019, *d* = 0.97; TPOJ1: *F*(1, 56) = 11.83, *p* = 0.001, *d* = 0.93] and ASD [STSdp: *F*(1, 48) = 4.48, *p* = 0.040, *d* = 0.86; TPOJ1: *F*(1, 48) = 9.43, *p* = 0.004, *d* = 0.71] participants, compared to control subjects. Across subjects, activation of TPOJ1 was driven strongly by dynamic, relative to static, faces [*F*(1, 75) = 57.45, *p* < 0.001] (Figure 3C) with no main effect of emotion. In contrast, activation of STSdp was larger for both emotional (vs. neutral) [*F*(1, 75) = 9.19, *p* = 0.003] and dynamic (vs. static) [*F*(1, 75) = 8.73, *p* = 0.004] faces. Further, deficits in both clinical groups were differentially greatest in response to dynamic emotional faces [*F*(2, 75) = 5.28, *p* = 0.025].

#### Amygdala

**FIGURE 4:**
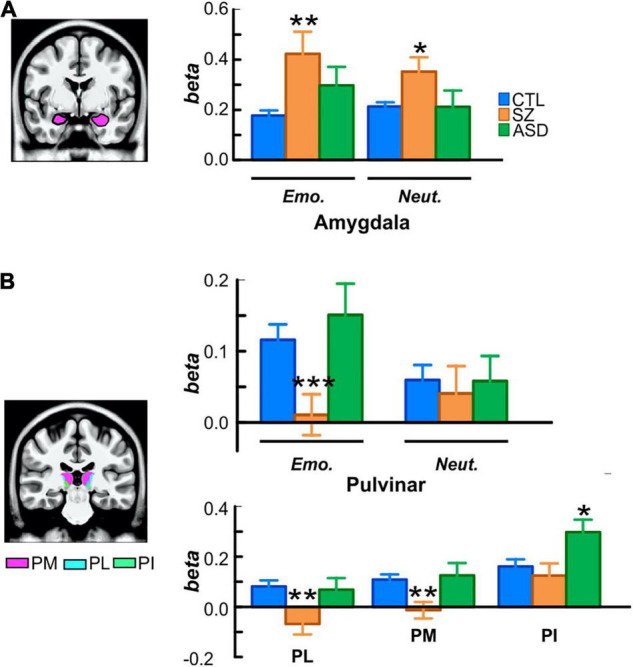
**(A)** Localization and activation of the amygdala in each participant group, superimposed on an MNI template volume. **(B)** Localization of the medial (pink; PM), lateral (cyan; PL), and inferior (green, PI) subdivisions of pulvinar. For each group, bar plots (top) are of beta values for emotional and neutral faces extracted from the whole pulvinar and (bottom) of mean betas from each subdivision. *p < 0.05; **p < 0.01; ***p < 0.001.

Compared to control subjects, overall activation of the amygdala (collapsed across face-motion and face-emotion) was significantly higher in Sz patients [*F*(1, 56) = 7.58, *p* = 0.008, *d* = 0.83]. Further, the interaction between group-membership and face-emotion was significant [*F*(1, 56) = 4.93, *p* = 0.030] reflecting greater group differences for emotional, relative to neutral, faces [*F*(1, 56) = 4.93, *p* = 0.030] (Figure 4A).

Compared to controls, activation of the amygdala was equivalent, overall, in ASD participants [*F*(1, 48) = 1.39, *p* = 0.244].

#### Pulvinar

Relative to controls, subcortical activation within PulN was reduced overall in Sz [*F*(1, 56) = 4.35, *p* = 0.042, *d* = 0.44]. This reduction was relatively greater in response to emotional faces as indexed by the significant interaction between face-emotion and group membership [*F*(1, 56) = 4.96, *p* = 0.030, *d* = 0.74] (Figure 4B, top).

In contrast, mean activation of the PulN did not differ, overall, between ASD and control participants [*F*(1, 48) = 0.260, *p* = 0.614].

The PulN was segmented into its lateral (PL), inferior (PI) and medial (PM) subdivisions. Across subdivisions, mean activation differed overall as a function of group membership [*F*(2, 75) = 4.91, *p* = 0.009]. In PL [*F*(1, 56) = 9.97, *p* = 0.003, *d* = 0.82] and PM [*F*(1, 56) = 10.35, *p* = 0.002, *d* = 0.84], activation was significantly reduced in Sz relative to controls. Neither PL [*F*(1, 48) = 0.068, *p* = 0.795] nor PM [*F*(1, 48) = 0.127, *p* = 0.723] activation distinguished between ASD and control groups (Figure 4B, bottom). A different pattern was observed in PI where activation was equivalent in Sz [*F*(1, 56) = 0.43, *p* = 0.513] compared to controls, but significantly enhanced in ASD [*F*(1, 48) = 6.42, *p* = 0.015, *d* = 0.71], reflecting a disorder-specific, double-dissociation of subnucleus activation patterns in Sz vs. ASD.

### Functional Magnetic Resonance Imaging Interrelationships

**FIGURE 5:**
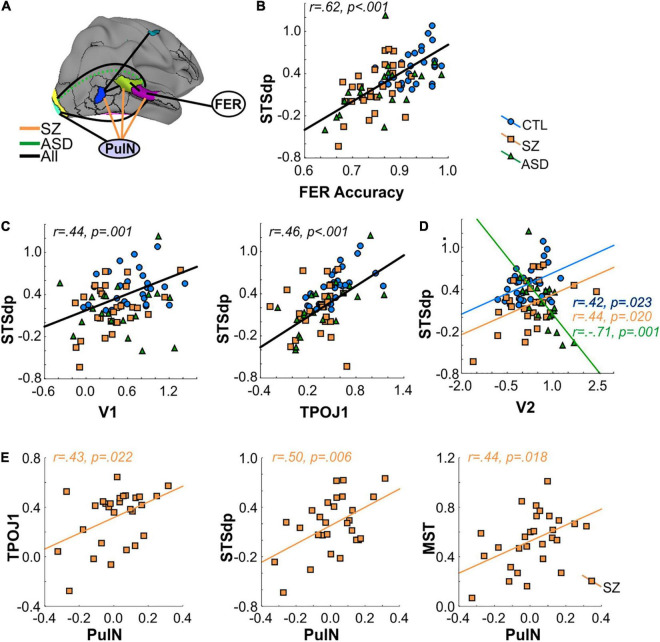
**(A)** Interrelationship between activated cortical regions, pulvinar (PulN) activation and face-emotion recognition (FER) accuracy. Black lines denote a significant relationship across all groups. Orange lines denote significant relationship only in SZ group. Dashed green line denotes a significant (negative) correlation in ASD group only. **(B)** Correlation between FER accuracy and mean activation (beta) of the STSdp parcel. In all cases, black regression lines signify the correlation was significant across groups. **(C)** Correlation between STSdp-V1 and STSdp-TPOJ1 activation. **(D)** Correlation between STSdp-V2 activation. In ASD participants, enhanced V2 was associated with reduced STSdp activation. The opposite relationship was obtained in SZ and CTL groups. **(E)** Scatterplots showing a significant and positive correlation between PulN activation and TPOJ1 (left), STSdp (center) and MST (right) in the SZ group alone.

The interrelationship between FER performance and cortical/subcortical activation patterns was assessed by ANCOVA. The results are summarized schematically in Figure 5A (see also Supplementary Table 2).

An initial omnibus analysis tested FER simultaneously against mean activation in all nine fMRI regions identified in the between-group fMRI analyses (seven cortical parcels, amygdala, PulN) (Supplementary Table 2A). This model tested the null hypothesis that no regions significantly predicted FER performance beyond the effect of group membership. The model incorporating these covariates (Adj. *R*^2^ = 0.49) was statistically superior to a model incorporating group membership alone [Adj. *R*^2^ = 0.35; *F*(9, 68) = 3.42, *p* = 0.0002] (Supplementary Table 2B) indicating that incorporation of these covariates significantly improved model fit.

The statistical contribution of the independent covariates was therefore considered in order to evaluate which regions contributed most to the overall model improvement. As expected, activation of the STSdp parcel most significantly predicted FER scores across participants [*F*(1, 66) = 16.61, *p* < 0.001]. Moreover, a model incorporating only STSdp as a covariate showed a model fit (Adj. *R*^2^ = 0.51; Supplementary Table 2C) similar to that of the more complex model incorporating all covariates. In this simpler model, the relationship between STSdp and FER was highly significant [*F*(1, 74) = 26.24, *p* < 0.001]. In all groups, greater activation of STSdp correlated with improved behavioral performance (covaried by age and IQ) (Sz: *r*_*p*_ = 0.46, *p* = 0.018; ASD: *r*_*p*_ = 0.53, *p* = 0.020; control: *r*_*p*_ = 0.41, *p* = 0.029) (Figure 5B). By contrast, no significant correlation was observed between FER and the other covariates in the analysis.

The relationship between the nine fMRI covariates was assessed in follow-up ANCOVAs run in stepwise fashion and including interactions with group membership in the model. As these were not independent tests of the overall null hypothesis, they were not considered to increase family-wise error regarding potential predictors of FER impairments across groups. Rather, the goal was to determine stepwise contributions to impaired STSdp activation, which was shown in the omnibus test to significantly predict FER across groups.

Mean activation of STSdp was predicted across groups by both V1 [*F*(1, 51) = 6.19, *p* = 0.016] and TPOJ1 [*F*(1, 51) = 6.77, *p* = 0.012] (Figure 5C). In turn, V1 was predicted by PulN activity [*F*(1, 54) = 8.80, *p* = 0.004], and TPOJ1 was predicted by MST [*F*(1, 57) = 18.29, *p* < 0.001], which also predicted FEF activation [*F*(1, 61) = 6.63, *p* = 0.012]. There were no further significant interrelationships across groups.

An interaction involving group membership, indicative of different slopes, was obtained between STSdp and V2 [*F*(2, 51) = 5.95, *p* = 0.005] such that in ASD participants enhanced activation of V2 was associated with diminished STSdp activity (*r*_*p*_ = −0.71, *p* = 0.001) whereas in controls (*r*_*p*_ = 0.42, *p* = 0.023) and Sz (*r*_*p*_ = 0.44, *p* = 0.020) there was an inverse relationship (Figure 5D).

Lastly, group-specific interactions involving subcortical (PulN) activity were also obtained. Specifically, in Sz patients only, activation of STSdp [*F*(2, 51) = 4.51, *p* = 0.016], TPOJ1 [*F*(2, 54) = 4.48, *p* = 0.016], and MST [*F*(2, 63) = 3.01, *p* = 0.047] were all significantly and positively associated with PulN activation (Figure 5E).

### Mediation Analyses

A more detailed analysis of subcortical activation patterns in Sz was carried out with exploratory mediation analyses involving specific PulN subdivisions and interrelated cortical regions. Based on known anatomical interrelationships within the early visual system (Bridge et al., 2016) as well as the regression analyses described above, three specific predictor-outcome paths were evaluated (V1-STSdp, MST-TPJO1 and TPOJ1-STSdp) with PL, PM, or PI as potential mediators. The results are detailed in Table 3.

**TABLE 3:** Results of mediation analyses testing whether a proposed causal effect of X (predictor) on Y (outcome) may be transmitted through a mediating (M) variable.

							95% CI		Std. Coeff.
		Path	B	SE	t	p	UL	LL	β
	V1-PL	a	0.420	0.120	3.650	0.001	0.180	0.660	0.580
*X* = *V1*	PL-STSdp	b	1.060	0.340	3.130	0.005	0.360	1.760	0.590
*Y* = *STSdp*	V1-STSdp	c	0.540	0.230	2.360	0.030	0.070	1.020	0.420
*M* = *PL*	V1-STSdp | PL	c’	0.100	0.250	0.400	0.694	−0.410	0.600	0.080
	Indirect	a*b	0.450	0.190	–	–	0.076	0.840	–
	TPOJ1-PM	a	0.467	0.124	3.752	0.001	0.211	0.723	0.590
*X* = *TPOJ1*	PM-STSdp	b	1.258	0.468	2.688	0.013	0.294	2.221	0.540
*Y* = *STSdp*	TPOJ1-STSdp	c	0.707	0.330	2.139	0.042	0.027	1.386	0.390
*M* = *PM*	TPOJ1-STSdp | PM	c’	0.120	0.369	0.325	0.748	−0.639	0.879	0.070
	Indirect	a*b	0.587	0.286	–	–	0.002	1.125	–
	PI-MST	a	0.424	0.147	2.884	0.008	0.122	0.726	0.490
*X* = *PI*	MST-TPOJ1	b	0.615	0.162	3.807	0.001	0.282	0.948	0.620
*Y* = *TPOJ1*	PI-TPOJ1	c	0.388	0.149	2.600	0.015	0.081	0.695	0.450
*M* = *MST*	PI-TPOJ1 | MST	c’	0.127	0.139	0.915	0.369	−0.159	0.414	0.150
	Indirect	a*b	0.261	0.118	–	–	0.129	0.596	–

For each mediation analysis, the coefficients (B), standard error (SE), t-statistic, p-value and lower (LL) and upper (UL) levels for the 95% confidence interval (CI) and standardized beta coefficient are given for the direct paths: (a) X and M; (b) M and Y; (c) X and Y; (c’) X and Y, conditional on M and for the indirect (a*b) effect. In the first analysis (top), activity from V1 and STSdp (pSTS) parcels are the predictor and outcome variables, respectively, mediated by lateral pulvinar (PL) activation. Middle section shows the path from TPOJ1 (TPJ) to pSTS, mediated by medial pulvinar (PM). Bottom section shows the relationship between inferior pulvinar (PI) and MST mediated by TPJ. *p < 0.05.

**FIGURE 6:**
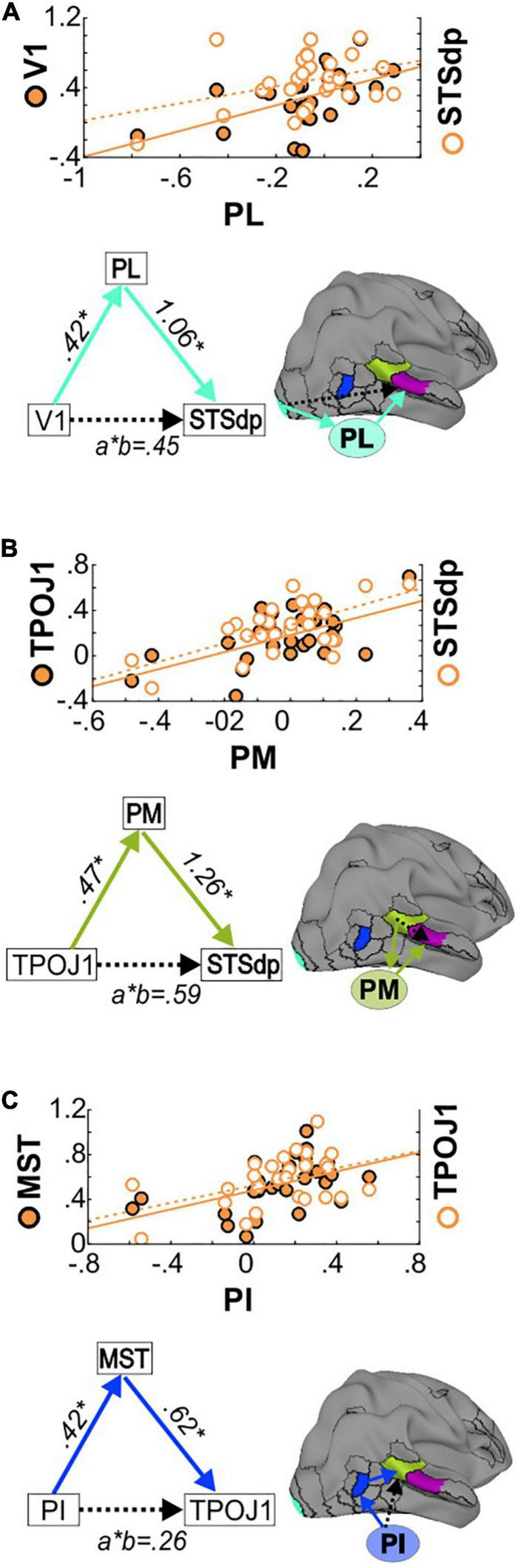
**(A)** (Top) Correlations between PL and V1 activation (left Y-axis, solid circles) and between PL and STSdp activation (right Y-axis, open circles) in SZ patients. (Bottom) Schematic of the proposed V1 → PL → STSdp path. Beta coefficients are given for the direct (a: V1-PL; b: PL-STSdp) and indirect (a*b) paths. **(B)** Correlations and TPOJ1 → PM → STSdp mediated path. **(C)** Correlations and PI → MST → TPOJ1 path.

Consistent with known anatomical projections of the lateral subdivision, activation of V1 significantly predicted mean PL activity (*p* = 0.001), which in turn predicted STSdp activation (*p* = 0.005) (Figure 6A). After controlling for PL activity, V1 was no longer associated with STSdp (*p* = 0.694), however, using a bootstrapping approach, the (unstandardized) coefficient for the indirect pathway from V1 to STSdp (V1 → PL → STSdp) was significant (CI: [0.08, 0.84]), consistent with full mediation.

In addition, a significant mediating effect of PM was observed between TPOJ1 and STSdp (TPOJ1 → PM → STSdp; CI: [0.002, 1.13]) (Figure 6B) and of MST between PI and TPOJ1 (PI → MST → TPOJ1; CI: [0.13, 0.60]) (Figure 6C). In both cases, after controlling for the proposed mediator the direct paths between were not significant, suggesting full mediation.

## Clinical Correlations

No significant correlations were observed between behavioral FER performance or cortical/subcortical activation patterns and medication dose (CPZ equivalents) in Sz patients (*p* > 0.15 for all). Functional activation strengths did not correlate with measures of general cognitive ability (PSI and IQ) in any group (*p* > 0.11 for all), however, in Sz (*r* = 0.378, *p* = 0.049) and control (*r* = 0.466, *p* = 0.044) participants, perceptual organization skill (POI) correlated with performance on the FER task as well as with STSdp activation (control: *r* = 0.539, *p* = 0.017; Sz: *r* = 0.399, *p* = 0.035).

## Discussion

Deficits in social cognition contribute disproportionately to impaired functional outcome across a range of neurocognitive disorders, including Sz and ASD. FER is an important component of these deficits and, in the visual system, depends upon coordinated function of both subcortical and cortical regions for processing of static and dynamic facial features. Here, we investigated cortical and subcortical correlates of FER impairments in adults with Sz and ASD using a dynamic/static FER task that engages motion-sensitive areas as well as traditional face-processing regions. In addition, we investigated the subcortical pathway to cortex involving PulN.

The primary findings of the study relate to the relative involvement of cortico-cortical vs. thalamo-cortical transmission paths underlying impaired FER in Sz. Traditionally, it was assumed that cortical regions showing intercorrelated activity mediate their joint activations primarily through direct cortico-cortical connections (Felleman and Van Essen, 1991; Scannell and Young, 1993). More recent models by contrast propose that connections are mediated primarily by successive loops between cortex and thalamus, with higher-tier thalamic regions such as PulN and dorsomedial nuclei generally interacting with posterior and anterior association regions, respectively (Sherman and Guillery, 1996; Llinas et al., 1998). Within PulN, discrete subnuclei interact with specific visual cortical regions (Bridge et al., 2016). This theory converges with anatomical studies showing reduced PulN volume and cell number in schizophrenia (Byne et al., 2002, 2007; Dorph-Petersen and Lewis, 2017), along with our recent observations of impaired PulN activation to simple visual stimuli in Sz (Martinez et al., 2018, 2019).

In the present study, activation deficits in Sz were observed within the HCP-MMP1.0 (Glasser et al., 2016) parcels comprising lower-tier visual regions including early visual and motion-sensitive cortex, along with higher-tier (multisensory) regions associated with FER.

Within STS two discrete parcels were activated by the task– STSdp and TPOJ1. Activation of the STSdp, in particular, showed uniquely greater activation to dynamic emotional faces which correlated with behavioral measures of FER, in accord with the prominent role of STS in face-emotion assessment (Haist and Anzures, 2017). In Sz, STSdp deficits intercorrelated with impairments in activation of both early visual cortical regions and PulN.

### Role of Pulvinar Subdivisions

PulN is divided into discrete anatomical subdivisions which mirror the dorsal/ventral stream distinction of visual cortex (Kaas and Baldwin, 2019) such that the more lateral regions (PL) project predominantly to primary visual cortex and ventral visual stream, whereas a subset of nuclei in the inferior subdivision (PI) project mainly to dorsal stream regions including motion-sensitive cortex (e.g., MST) (Arcaro et al., 2015; Tamietto and Morrone, 2016; Kaas and Baldwin, 2019). The medial subdivision (PM) is primarily connected with multimodal sensory association areas as well as prefrontal and cingulate cortices and has been tied to emotion processing (Homman-Ludiye and Bourne, 2019).

In the present study, in addition to intercorrelated cortical activation deficits, we observed correlations between cortical regions and PulN subnuclei. We therefore conducted a series of mediation analyses to evaluate underlying pathways.

An initial analysis evaluated the potential pathways underlying intercorrelated deficits between V1 and STS in the schizophrenia group. A significant indirect pathway from V1→PL→STSdp was observed, in support of indirect mediation by PL. Consistent with mediation by PM, the intercorrelation between the STS parcels (TPOJ1 and STSdp) was not significant once an indirect path *via* PM was modeled.

In contrast, PI appeared to mediate its effects *via* MST. Of note, unlike PL and PM, activation of PI was relatively intact in patients. Given that PI receives much of its driving inputs from neurons in the superior colliculus (Kaas and Baldwin, 2019), this finding is suggestive of unimpaired input *via* the retinotectal system. By contrast, the observed deficits in V1 are consistent with impaired input *via* the geniculostriate visual pathway (Martinez et al., 2008, 2019).

Although anatomical abnormalities in PulN are well-documented in schizophrenia (Dorph-Petersen and Lewis, 2017; Huang et al., 2020), the functional consequences of these abnormalities have, to date, remained relatively unexplored. Here, we provide evidence that impairments in visual PulN function significantly undermines visual processing required for effective face processing. In specific, deficits in PL function may mediate effects of impaired V1 activation, which, in turn likely reflects impaired magnocellular input to cortex. In addition, impaired PM activation mediated impaired input from more posterior (TPOJ1) to mid-STS (STSdp) regions, suggesting that accumulating deficits across successive cortico-pulvinar loops may lead to the large effect-sized deficits in FER-related reduced STS activation in Sz.

### Comparison to Autism Spectrum Disorders

While deficits in social cognition are a prominent component of Sz, they are not unique to the disorder. In particular, we (Martinez et al., 2019) and others (Couture et al., 2010; Sasson et al., 2016) have reported FER deficits in adult ASD subjects that are as severe as those observed in Sz, despite the much higher level of overall function. Consistent with these prior results, adult ASD participants in the present study showed FER and STS activation deficits that were similar to those of Sz, supporting a role for STS as a common mediator of FER dysfunction across disorders.

Despite the similar STS impairments, ASD participants showed markedly different patterns of disturbance within early visual regions. In contrast to Sz patients, markedly increased responses and an opposite slope of the relationship between V2 and STSdp activation were observed in the ASD group. Activation within other task-activated visual regions, including V1 and MST, was unaffected in ASD, echoing our recent study in which response amplitudes were also normal within V1, but increased in early visual and dorsal visual regions (Martinez et al., 2019). Similar visual hypo/hyper activation patterns in Sz vs. ASD have been observed in both fMRI (reviewed in Samson et al., 2012; Taylor et al., 2012) and electrophysiological (Martinez et al., 2018, 2019; Shah et al., 2018; Kovarski et al., 2019) studies, supporting the concept that dysregulation of the early visual system may undermine later stages of visual processing.

Patterns of amygdalar and subcortical activation also distinguished between ASD and Sz participants. In the amygdala, activation was elevated overall in Sz (but not ASD), as reported previously (reviewed in Dugre et al., 2019), possibly reflecting abnormal salience attribution to neutral stimuli (Holt et al., 2006; Gur et al., 2007; Premkumar et al., 2008), heightened anxiety (Du and Grace, 2016) and/or paranoid ideation (Pinkham et al., 2015).

In contrast, whereas PulN activity was markedly reduced in Sz, activation of the inferior PulN subdivision was significantly elevated in ASD participants, in line with findings from our previous studies (Martinez et al., 2018, 2019) and those of others (Zurcher et al., 2013; Hadjikhani et al., 2017; Martinez et al., 2019). Although the source of the increased activation in ASD is not known, a parsimonious explanation would be hyperactivity of the subcortical retino-collicular pathway, which provides preferential input to PI (Kaas and Baldwin, 2019) and which, in turn, acts like a driver to V2 (de Souza et al., 2020). In humans, this system typically weakens with age as the retinogeniculate system increases in functionality (reviewed in Bourne and Morrone, 2017). Abnormal persistence of this system into adulthood could thus underlie the activation disturbance pattern observed in ASD.

Regardless of underlying pathophysiology, these findings support the concept that dysregulation of the early visual system, whether in the direction of increased or decreased activation, may undermine later stages of visual processing and further highlight the importance of sensory processing abnormalities to the pathophysiology of social cognitive impairment across neuropsychiatric disorders.

### Limitations

Despite our differential findings, some limitations must be considered. First, Sz participants were receiving antipsychotic medication which may have impacted measures on brain activity. We did not observe any correlations with medication dose, however, this issue could be best addressed in future studies involving medication-naïve patients. Moreover, we note that the overall sample size remains small, and the findings need to be replicated in an independent sample. Additionally, we did not track fixation locations either in the scanner or during behavior. Thus, we do not know if activation failures relate to inability to process information, or simply from differential facial scanning approaches. Lastly, Sz participants had lower IQ and were older than controls or ASD participants, although correcting for IQ and age did not diminish the findings.

## Conclusion

In summary, higher cortical (e.g., STS) contributions to impaired FER have been extensively documented in Sz and ASD (Fernandes et al., 2018), but early visual and subcortical contributions have been evaluated to only a limited degree. Here, we demonstrate significant but opposite abnormalities of circuits centered on PulN in Sz vs. ASD that correlate with impaired STS activation, which in turn correlated across groups with impaired FER. These findings highlight the importance of close integration between subcortical and cortical visual processing pathways and the potential breakdown of this tight coordination in Sz and ASD. Further, the findings reaffirm that similar behavioral deficits (e.g., impairment in social cognition) do not necessarily imply convergent pathophysiological mechanisms, and that physiological measures may be useful for guiding etiological and interventional studies in neuropsychiatry.

## Data Availability Statement

The original contributions presented in the study are included in the article/Supplementary Material, further inquiries can be directed to the corresponding author.

## Ethics Statement

The studies involving human participants were reviewed and approved by the Nathan Kline Institute for Psychiatric Research, Institutional Review Board. The patients/participants provided their written informed consent to participate in this study.

## Author Contributions

AM wrote the first draft of the manuscript. PG and DCJ contributed to the conception and designed of the study. RT and GS were involved in subject recruitment and characterization. ED, PS, PL, and GP reviewed and edited drafts of the manuscript. DB analyzed parts of the data. DM conducted the mediation analyses. All authors contributed to the article and approved the submitted version.

## Conflict of Interest

DCJ holds equity in Glytech, AASI, and NeuroRx; is part of the scientific advisory board for NRx pharma; and received consultant payments from Concert, Lundbeck, Phytec, Autifony, SK Life Sciences, Biogen, Cadence, Boehringer-Ingelheim, and Pfizer; DCJ has also received research support from Cerevance unrelated to this project.

## Publisher’s Note

All claims expressed in this article are solely those of the authors and do not necessarily represent those of their affiliated organizations, or those of the publisher, the editors and the reviewers. Any product that may be evaluated in this article, or claim that may be made by its manufacturer, is not guaranteed or endorsed by the publisher.

## Footnotes

1
https://afni.nimh.nih.gov

2
https://www.ibm.com/products/spss-statistics
