# Supplementary material for: Disease-Specific Contribution of Pulvinar Dysfunction to Impaired Emotion Recognition in Schizophrenia
Source: Front Behav Neurosci. 2022 Feb 14;15:787383. doi: 10.3389/fnbeh.2021.787383 (PMC8883821; doi:10.3389/fnbeh.2021.787383)
Supplement: Supplementary file 1 [file Table_1.docx]

**A. Tests of Between-Subjects Effects**

| **Source** | **Type III Sum of Squares** | **df** | **Mean Square** | **F** | **Sig.** |
| --- | --- | --- | --- | --- | --- |
| **Intercept** | **8.001** | **1** | **8.001** | **6.970** | **.010** |
| **Age** | **1.951** | **1** | **1.951** | **1.699** | **.196** |
| **IQ** | **.474** | **1** | **.474** | **.413** | **.522** |
| **Group** | **.244** | **2** | **.122** | **.106** | **.899** |
| **Error** | **83.793** | **73** | **1.148** |  |  |

**B. Multivariate Tests^a^**

| **Effect** | **Value** | | **F** | **Hypothesis df** | **Error df** | **Sig.** |
| --- | --- | --- | --- | --- | --- | --- |
| **Parcels** | **Wilks' Lambda** | **.408** | **1.708** | **34.000** | **40.000** | **.052** |
| **Parcels * Age** | **Wilks' Lambda** | **.543** | **.990** | **34.000** | **40.000** | **.508** |
| **Parcels * IQ** | **Wilks' Lambda** | **.504** | **1.159** | **34.000** | **40.000** | **.325** |
| **Parcels * Group** | **Wilks' Lambda** | **.159** | **1.776** | **68.000** | **80.000** | **.007** |

**a. Design: Intercept + Age + IQ + Group**

**Within Subjects Design: Parcels**

**Supplementary Table 1: A)** Omnibus repeated-measures ANOVA across all parcels within the 35-parcel mask with participant age and IQ entered as covariates. This test evaluated the null hypothesis that there were no significant between-group differences in cortical activation across parcels**. B)** Results of multivariate tests. The null hypothesis was falsified based on a significant Group X Parcel interaction, indicating that activation in some, but not all parcels, differed across groups.

**A. Tests of Between-Subjects Effects**

**Dependent Variable: FER**

| **Type III Sum of**  **Source Squares** | | **df** | **Mean Square** | **F** | **Sig.** | **Partial Eta Squared** |
| --- | --- | --- | --- | --- | --- | --- |
| **Corrected Model** | **.368^a^** | **11** | **.033** | **7.753** | **.000** | **.564** |
| **Intercept** | **4.743** | **1** | **4.743** | **1099.984** | **.000** | **.943** |
| **V1** | **.003** | **1** | **.003** | **.716** | **.400** | **.011** |
| **V2** | **2.038E-7** | **1** | **2.038E-7** | **.000** | **.995** | **.000** |
| **FFC** | **.005** | **1** | **.005** | **1.046** | **.310** | **.016** |
| **MST** | **.005** | **1** | **.005** | **1.126** | **.293** | **.017** |
| **TPOJ1** | **.000** | **1** | **.000** | **.033** | **.856** | **.001** |
| **STSdp** | **.072** | **1** | **.072** | **16.611** | **.000** | **.201** |
| **FEF** | **.000** | **1** | **.000** | **.093** | **.761** | **.001** |
| **Pulvinar** | **.002** | **1** | **.002** | **.578** | **.450** | **.009** |
| **Amygdala** | **.002** | **1** | **.002** | **.567** | **.454** | **.009** |
| **Group** | **.092** | **2** | **.046** | **10.689** | **.000** | **.245** |
| **Error** | **.285** | **66** | **.004** |  |  |  |
| **Total** | **53.324** | **78** |  |  |  |  |
| **Corrected Total** | **.652** | **77** |  |  |  |  |

**a. R Squared = .564 (Adjusted R Squared = .491)**

**b. Computed using alpha = .05**

**B. Tests of Between-Subjects Effects**

**Dependent Variable: FER**

| **Type III Sum of**  **Source Squares** | | **df** | **Mean Square** | **F** | **Sig.** | **Partial Eta Squared** |
| --- | --- | --- | --- | --- | --- | --- |
| **Corrected Model** | **.239^a^** | **2** | **.120** | **21.729** | **.000** | **.367** |
| **Intercept** | **50.487** | **1** | **50.487** | **9167.606** | **.000** | **.992** |
| **Group** | **.239** | **2** | **.120** | **21.729** | **.000** | **.367** |
| **Error** | **.413** | **75** | **.006** |  |  |  |
| **Total** | **53.324** | **78** |  |  |  |  |
| **Corrected Total** | **.652** | **77** |  |  |  |  |

**a. R Squared = .367 (Adjusted R Squared = .350)**

**b. Computed using alpha = .05**

**C. Tests of Between-Subjects Effects**

**Dependent Variable: FER**

| **Type III Sum of**  **Source Squares** | | **df** | **Mean Square** | **F** | **Sig.** | **Partial Eta Squared** |
| --- | --- | --- | --- | --- | --- | --- |
| **Corrected Model** | **.347^a^** | **3** | **.116** | **28.106** | **.000** | **.533** |
| **Intercept** | **24.897** | **1** | **24.897** | **6042.162** | **.000** | **.988** |
| **STSdp** | **.108** | **1** | **.108** | **26.236** | **.000** | **.262** |
| **Group** | **.101** | **2** | **.050** | **12.217** | **.000** | **.248** |
| **Error** | **.305** | **74** | **.004** |  |  |  |
| **Total** | **53.324** | **78** |  |  |  |  |
| **Corrected Total** | **.652** | **77** |  |  |  |  |

**a. R Squared = .533 (Adjusted R Squared = .514)**

**b. Computed using alpha = .05**

**Supplementary Table 2 A)** Omnibus ANCOVA in which all nine fMRI regions were evaluated simultaneously versus FER. **B)** ANOVA model in which only group was considered as a predictor of performance on the FER task**.** **C)** ANCOVA including only STSdp activation along with group membership as predictors of FER.
